# ID4: a new player in the cancer arena

**DOI:** 10.18632/oncotarget.108

**Published:** 2010-05-01

**Authors:** Stefania Dell'Orso, Federica Ganci, Sabrina Strano, Giovanni Blandino, Giulia Fontemaggi

**Affiliations:** ^1^*Translational Oncogenomics Unit, Regina Elena Cancer Institute, 00144-Rome, Italy.*; ^2^*Rome Oncogenomic Center (ROC), Regina Elena Cancer Institute, 00144-Rome, Italy.*; ^3^*Molecular Chemoprevention Group, Scientific Direction, Regina Elena Cancer Institute, 00144-Rome, Italy.*; ^4^*General Pathology Section, Department of Clinical and Experimental Medicine, Perugia University, Perugia, Italy.*

**Keywords:** Id family, mutant p53, gain of function, Id4, chemoresistance

## Abstract

Id proteins (Id-1 to 4) are dominant negative regulators of basic helix-loop-helix transcription factors. They play a key role during development, preventing cell differentiation while inducing cell proliferation. They are poorly expressed in adult life but can be reactivated in tumorigenesis. Several evidences indicate that Id proteins are associated with loss of differentiation, unrestricted proliferation and neoangiogenesis in diverse human cancers. Recently, we identified Id4 as a transcriptional target of the protein complex mutant p53/E2F1/p300 in breast cancer. Id4 protein binds, stabilizes and enhances the translation of mRNAs encoding proangiogenic cytokines, such as IL8 and GRO-alpha, increasing the angiogenic potential of cancer cells. We present here an overview of the current experimental data that links Id4 to cancer. We provide evidence also of the induction of Id4 following anticancer treatments in mutant p53-carrying cells. Indeed, mutant p53 is recruited to a specific region of the Id4 promoter upon DNA damage. Our findings indicate that Id4, besides its proangiogenic role, might also participate in the chemoresistance associated to mutant p53 proteins exerting gain of function activities.

## INTRODUCTION

The basic-helix-loop-helix (bHLH) family of transcription factors has been shown to play a key role in the differentiation processes of a number of cell lineages. These proteins contain an HLH domain, which mediates homo-and hetero-dimerization, plus an adjacent DNA-binding region rich in basic amino acids. The bHLH proteins bind to a DNA sequence known as E-box (CANNTG). There are two major categories of bHLH. Class A are ubiquitously expressed proteins such as those encoded by the differently spliced transcripts of E2A (E12, E47, E2-5), E2-2 and HEB genes [1]. Class B comprises tissue-specific bHLH proteins that form heterodimers with a partner from the ubiquitously expressed class A family [2].

A sub-class of HLH genes, which lacks the basic DNA-binding domain, is known as Inhibitors of DNA binding (Id) genes. The proteins encoded by these genes act as dominant-negative regulators of bHLH proteins by forming inactive heterodimeric complexes. In mammals there are four known Id gene family members known as Id1, Id2, Id3 and Id4. The best characterized Id protein interaction is with the ubiquitously expressed bHLH E proteins (E2-2, E12, E47), which heterodimerize with tissue-specific bHLH proteins, such as MyoD (in muscle) and NeuroD (in nerves).

The identity between the HLH regions of Id proteins is very high, while the remaining regions of the proteins are not conserved. A study from Kieviz and Cabrele [3] reported that the N- and C-terminal fragments of Id proteins do not adopt a helical conformation, with the exception of Id4 fragment 27-64. This helix propensity is dictated by the presence of an Ala-rich motif between residues 39 and 57. It can be hypothesized that Id4 might exert unique functions through this structural feature. Despite the high similarity in the HLH domain, the Id proteins bind different targets with different affinities; for example Id2 is the only Id family member that recognizes the retinoblastoma protein [4, 5].

Id proteins were described initially as inhibitors of differentiation and more recently as regulators of cell cycle progression, senescence, apoptosis and tumorigenesis [6-9]. Id proteins play a critical role in promoting the progression through the S-phase of the cell cycle in cell culture cells. Id1, Id2 and Id3 have been shown to interact with cell cycle regulatory molecules [10]. Indeed, they negatively regulate the expression of cyclin D1, p16Ink4a and p21CIP1/WAF1 [11, 12, 13]. The expression and function of Id proteins need to be strictly controlled to ensure the correct timing of cell cycle exit and differentiation. The role of Id2 has been well characterized in this regard. Indeed Id2 physically interacts with the tumor suppressor protein Retinoblastoma (pRb). Genetic analyses have shown that pRb restrains Id2 activity during development to prevent ectopic proliferation and apoptosis and to promote differentiation. The absence of functional pRb leads to a gain of Id2 activity and inappropriate sequestration of E proteins, causing a block in the cell cycle exit and differentiation. Loss of Id2 partially compensates loss of function of pRb. This results in the block of differentiation of the nervous system and hematopoietic compartments [5].

**Fig. 1:**
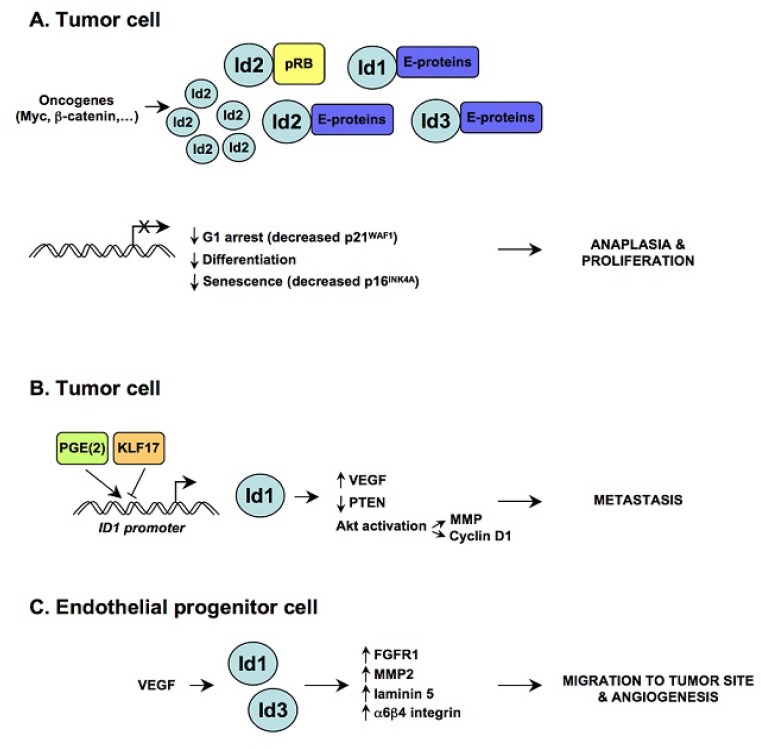
Deregulated Id signaling may promote multiple attributes of malignancy (A) Increased levels of Id proteins have been reported in cancer cells and their expression is frequently governed by activated oncogenes (such as Myc and beta-catenin that control Id2 expression). In non transformed cells Rb restrains Id2 function by direct interaction [4, 5]; during tumorigenesis Id2 protein levels increase and overcome Rb control resulting in unresctriced proliferation. Id proteins accumulation also leads to inhibition of the bHLH factors (E-proteins and ETS) responsible for cell cycle exit, differentiation and senescence [11, 13, 46-50], causing anaplasia. (B) Id1 expression in breast cancer cells is induced by cyclooxygenase-2-derived prostaglandin E2 (promoting metastasis) while is repressed by KLF17 (a metastatic suppressor) [51, 52]. Id1 expression may negatively regulate PTEN, leading to Akt activation, and drives tumor reinitiation during breast cancer metastasis [53-56]. (C) In endothelial progenitor cells Id1 and Id3 maintain the expression of FGFR1, MMP2, laminin 5 and alpha 6-beta 4 integrin [57], thereby enabling the mobilization of EPC from the bone marrow to the site of the tumor in response to circulating cytokines [17, 18].

As outlined above, Id proteins are required for proper development and differentiation. However, the expression of Id proteins, which is very low in adult tissues, can be reactivated in human cancers. It has been proposed that deregulated Id signaling may promote multiple attributes of malignancy (summarized in Figure 1), like unrestricted proliferation, loss of differentiation (anaplasia), invasiveness and neoangiogenesis [9, 14]. Elevated levels of Id proteins have been reported in several malignancies (carcinoma, squamous cell carcinoma, adenocarcinoma, neural tumors, melanoma, sarcoma, seminoma and leukemia) [9]. In some cases, high levels are associated with tumor stage/grade and with prognosis. Analysis of genetic alterations of Id genes in human tumors has found no mutations. This may indicate that Id genes are not common oncogenes. Nevertheless, the overexpression of Id genes in cancer is frequently governed by *bona fide* oncogenes, such as MYC driven Id2 expression in neuroblastoma cells [5, 15] and beta-catenin driven Id2 expresion in colon cancer cells [16]. The role of Id proteins in cancer seems to rely on activity carried out in different cell compartments. Id protein expression is upregulated in the tumor itself, leading to enhanced cell proliferation and inhibition of senescence [9]. Genetic studies on Id1 and Id3 knock-out mice have shown that Ids are expressed in endothelial progenitor cells (EPC) and are required for EPC mobilization from the bone marrow during pathological tumor-induced neoangiogenesis [8]. The expression of Ids remains high in tumor neovasculature [17] and drives the “angiogenic switch” required for the progression from micro- to macro-metastases [18].

### Id4 in neural development and cancer

Id4 is the least studied member of the Id family of proteins. Several lines of evidence suggest that Id4 plays an important role in the nervous system, and in particular in oligodendrocyte development. Id4 is expressed in oligodendrocyte precursor cells and may control the timing of oligodendrocyte differentiation. Enforced expression of Id4 *in vitro* stimulates proliferation and blocks differentiation of oligodendrocyte precursor cells [19]. Id4 was recently found to directly interact with bHLH, OLIG1 and OLIG2 in neural progenitor cells. It also mediates the inhibitory effects of bone morphogenetic protein-4 (BMP-4) on oligodendroglial differentiation that leads to astrocytic differentiation [20]. Studies on knock-out mice revealed that Id4 is required for normal brain size and regulates neural stem cells proliferation and differentiation [21]. In particular, Id4 regulates lateral expansion of the proliferative zone in the developing cortex and hippocampus. Since Id4 is required for the normal G1/S transition in early cortical progenitors, the absence of its expression compromises the proliferation of stem cells in the ventricular zone [21].

It has been established that developmental regulators play a direct role in driving aspiring cancer cells towards a malignant phenotype, and contribute to the conferring of stem-like cell properties, including robust renewal potential [22]. Enforced Id4 expression can drive malignant transformation of primary murine Ink4a/Arf−/− astrocytes, thereby highlighting the role of Id4 in controlling the “stemness” of neural cells during development of the central nervous system [23]. Id4 increases the levels of both cyclin E (that leads to a hyperproliferative state) and Jagged1 to drive astrocytes into a neural stem-like cell state. Id4 mRNA levels were found to have increased in human glioblastoma multiforme (GBM) when compared to normal brain tissue. Interestingly, the analysis of Id4 protein expression in human GBM specimens evidenced that the majority of Id4-positive cells resides near the vasculature, a location postulated to be the niche for brain tumor stem cells [24]. Conversely to that observed in brain tumors, reduced Id4 expression due to promoter hypermethylation was observed in gastric and colorectal carcinomas, indicating a possible role of Id4 in tumor suppression (see Table 1). Id4 promoter was also found hypermethylated in a variety of other malignancies, such as leukemia, prostate cancer and breast cancer (summarized in Table 1).

### Id4 in breast cancer

The analysis of Id4 expression in breast cancer has lead to seemingly controversial findings. This might be due to the scarcity of available information regarding the role of Id4 in tumorigenesis. Furthermore, each breast cancer subtype represents a distinct pathology characterized by specific cytogenetic and molecular alterations, proliferation rate, metastatic potential and response to conventional anticancer treatments.

*In situ* hybridization analysis of normal breast epithelium and carcinoma has shown that Id4 is expressed only in estrogen receptor negative (ER-) tissues [25]. ER-positive (ER+) cells are negative for Id4 expression both in normal epithelium and carcinoma. Following these findings a tumor suppressor role for Id4 in human breast has been proposed. Analysis of the methylation status of Id4 promoter in breast cancer cell lines and tissues has indicated that hypermethylation is a frequent event and is associated with an increased risk of lymph node metastasis [26, 27]. To date the molecular mechanisms underlying the tumor suppressor activity of Id4 have not been characterized.

On the contrary, Beger and colleagues [28] have proposed a positive role for Id4 in mammary and ovarian tumorigenesis. The modulation of Id4 expression in breast and ovarian cancer cell lines resulted in inversely regulated expression of BRCA1. An increase of Id4 expression was associated with the ability of ovarian (PA-1) and breast (SKBr3) cancer cells to exhibit anchorage-independent growth, while its depletion determined morphological change to a large and flat epithelial phenotype. The expression of ID4 and BRCA1/ER inversely correlated in sporadic breast cancers [29]. Turner et al. [30] have reported that high expression of Id4 mRNA is present in basal-like breast cancer (expressing cytokeratines 5/6) when compared to matched non-basal controls, and Id4 expression correlates to low levels of BRCA1 mRNA. Additional proof of the active role of Id4 in breast tumorigenesis has been provided by Shan et al. [31] who found elevated nuclear expression of Id4 protein in mammary rat carcinoma compared to adenoma and normal tissue. Id4 protein nuclear staining in carcinomas was also positively correlated with proliferation, invasiveness and tumor weight. Enforced Id4 expression caused an increase in colony growth in soft agar [31].

**Table 1:** Id4 modulation in cancer

Kind of modulation	Kind of analysis	Tumor type	Reference
Nuclear localization in cancer vs cytoplasmic localization in spermatogonia	protein	Seminoma	[59]
Upregulation associated to amplification at 6p22.3	mRNA	Bladder	[60]
Hypermethylation	promoter DNA	Gastric adenocarcinoma	[61]
Hypermethylation Downregulation	promoter DNA protein	Colorectal carcinoma	[62]
Hypermethylation	promoter DNA	Colorectal adenocarcinoma	[63]
Downregulated in low grade cancer vs hyperplasia Upregulated in high grade vs low grade cancer	protein	Prostate	[64]
Downregulated	mRNA	Prostate	[65]
Hypermethylation	promoter DNA	Leukemia	[66]
Hypermethylation	promoter DNA	Lymphoma	[67]
Downregulated	protein	Breast	[68]
Upregulated	protein	Breast (rat)	[31]
Hypermethylation	promoter DNA	Breast	[27]
Upregulated in basal-like cancer vs non-basal-like cancer	mRNA	Breast	[30]
Hypermethylation	promoter DNA	Breast	[26]
Upregulated in p53-expressing cancer	protein	Breast	[37]
Upregulated in cancer vs normal brain	mRNA	Glioblastoma multiforme (GBM)	[23]
Upregulated in cancer vs adjacent normal tissue	protein	Small cell lung cancer	[69]
Hypermethylation	promoter DNA	Cholangiocarcinoma	[70]

### Id4 and p53 mutations

Half of all human cancers bear *TP53* mutations. Most of the p53 alterations are missense mutations, often within the conserved DNA binding core domain of the protein. The resulting proteins display a marked heterogeneity in terms of loss of structure and function. Several evidences demonstrate that a subset of p53 mutant proteins exert gain of function activity, thereby actively participating in tumorigenesis [32]. Mutant p53 has been shown to increase cellular resistance to anticancer treatments and to contribute to genomic instability by abrogating the mitotic spindle checkpoint, consequently facilitating the generation of aneuploid cells [33, 34]. Mutant p53 knock-in mice have a higher frequency of solid tumours with a high potential for metastasis, a feature not seen in p53 knock-out animals [35, 36].

We have recently shown that mutant p53 proteins specifically induce Id4 expression in experimental cell systems [37]. Moreover, Id4 protein expression is enriched in breast cancer tissues showing p53 overexpression, often correlated to a mutation in the coding sequence of *TP53* gene that confers a high level of stability to the protein. We found that Id4 is expressed in 44% of the breast cancer specimens analyzed (186 patients). As already mentioned, Id4 positivity is increased in the p53 overexpressing population (p53+), where it reaches 60%, compared to the p53-negative population (38%); this phenomenon is even more marked in the HER2 subtype (54 specimens), where Id4 is expressed in nearly 80% of p53+ cases, compared to 40% of the p53- cases [37]. HER2 overexpressing breast cancer subtype presents very high frequency of *TP53* mutations, like also cancers presenting a basal phenotype [38]. The small number of basal-like tissues examined in our study did not permit obtaining significant information about the potential correlations existing between Id4 and p53 expressions in this BC subtype. The interrogation of public gene expression data repositories (www.oncomine.org) [39] for Id4 mRNA expression in breast cancers revealed that high levels of Id4 transcript are present in basal-like versus nonbasal breast cancers in various studies (data not shown).

**Fig. 2:**
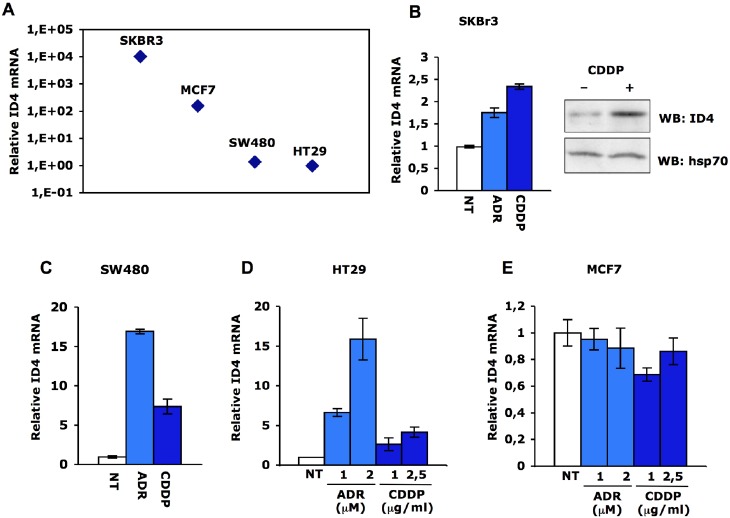
ID4 mRNA is increased in response to DNA damage **(A)** qRT-PCR analysis of ID4 expression was performed in parallel on SKBr3, MCF7, SW480 and HT29 cells. Relative ID4 mRNA levels were calculated by normalization for the amount of GAPDH transcript present in the RNA preparations. ID4 and GAPDH expressions were measured by real-time PCR using TaqMan assays (Applied Biosystems). **(B-C)** qRT-PCR analysis of ID4 expression in SKBr3 and SW480 following treatment with cisplatin (1μg/mL) or adriamycin (1μM) for 36 hours. ID4 protein expression in SKBr3 cells was evaluated by western blotting after 36h treatment with cisplatin (1μg/mL) using rabbit polyclonal anti-ID4 (Santa Cruz). **(D-E)** qRT-PCR analysis of ID4 expression in HT29 and MCF7 cells treated or not with the indicated amounts of adriamycin or cisplatin for 36h.

Further characterization performed in breast cancer cell lines enabled us to show that the transcriptional transactivation of Id4 promoter is exerted by the complex mutant p53/E2F1/p300 [37]. The net biological output of the transcriptional activation of Id4 gene by mutant p53 is the increase of the angiogenic potential of mutant p53-carrying tumor cells (see Figure 5). The binding of Id4 protein to the mRNAs of pro-angiogenic factors like IL8 (CXCL8) and GRO-alpha (CXCL1), that results in an increased stability and a higher rate of translation of these transcripts, explains the proangiogenic effects of Id4 transactivation. In parallel to these findings, obtained in cell lines, the staining of 110 breast cancers for the CD31 blood vessels marker revealed higher microvessel density in the Id4-positive population than that in Id4-negative [37]. Significantly, the most expressed cytokines in HER2 tumors are IL8 and GRO-alpha [40], cytokines which are also induced by the transcriptional axis mutant p53 and Id4.

We hypothesize that Id4 displays tumor suppressor functions in ER+ breast tumors where it is frequently inactivated by promoter hypermethylation. However, Id4 displays oncogenic activities in the context of breast cancer cells expressing mutant p53, which are mainly ER- [41, 42]. Thus, mutant p53-carrying cells express proteins required for the pro-tumorigenic function of Id4, such as factors that enable Id4 binding to proangiogenic target mRNAs. The expression profiling of breast cancer tissues with known p53 status has revealed that tumors with wild-type or mutated p53 are distinguished by pervasive molecular differences [42]. It is therefore likely that many unique players are present in mutant p53-carrying tissues.

### Id4 expression is induced by mutant p53 in response to DNA damage

While studying the dependency of Id4 on mutant p53 we sought to investigate whether Id4 expression is modulated by mutant p53 in response to commonly used anticancer drugs, such as adriamycin and cisplatin. The majority of the mutant p53 target genes so far identified are indeed modulated in response to anticancer agents, thereby providing a molecular basis for increased chemoresistance of tumors carrying *TP53* mutations. Recent findings by Di Agostino et al. [43] have shown that mutant p53 transactivates cell cycle regulatory genes in response to treatment with chemotherapeutic agents, thereby providing molecular-based insights into the aberrant regulation of cell cycle in tumor cells.

We first analyzed Id4 expression of SKBr3 and MCF7 breast cancer cells, carrying mutant p53R175H and wt-p53, respectively, and of SW480 (colon) and HT29 (colorectal) adenocarcinoma cells, carrying mutant p53R273H/P309S and p53R273H, respectively. SKBr3 (ER-) cells express high levels of Id4 while MCF7 (ER+) cells display 60-folds lower Id4 levels than SKBr3 (Fig. 2A). These findings correspond with the previously reported observation that Id4 expression is inversely correlated to ER expression [25]. Id4 expression is rather low in colorectal cancer cells (Fig. 2A). This parallels with Id4 promoter methylation as previously shown by Umetani et al. [27].

Next, we assessed Id4 expression upon cisplatin or adriamycin treatment. Id4 transcript is strongly induced upon DNA damage in SW480 and HT29 cells (Fig. 2C-D), while its induction is less pronounced in SKBr3 cells (Fig. 2B). Id4 expression is not induced in wt-p53 MCF7 breast cancer cells in response to cisplatin or adryamicin (Fig. 2E).

**Fig. 3:**
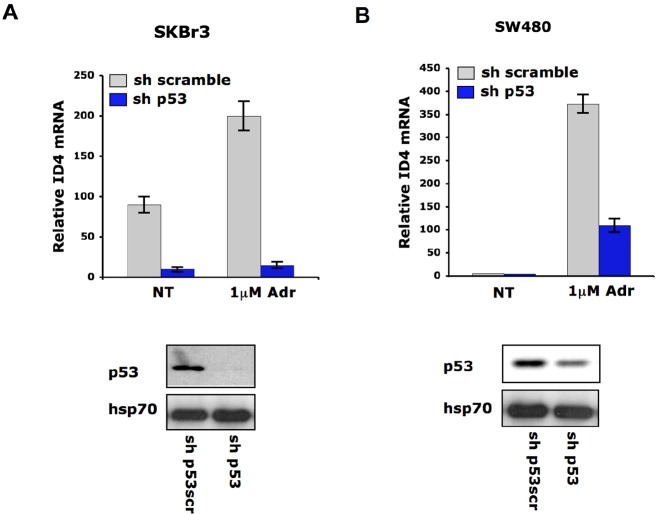
Mutant p53 mediates Id4 mRNA induction after DNA damage. qRT-PCR analysis of ID4 expression was performed in SKBr3 **(A)** and SW480 **(B)** cells whose p53 expression was depleted (sh-p53) and control cells (sh-scramble) upon treatment with adriamycin (1μM) for 36h. For p53 interference cells were transfected with pRS-p53-scramble and pRS-p53 plasmids and transfected cells were selected with puromycin. Mutant p53 protein expression of stably interfered SKBr3 and SW480 polyclonal populations was evaluated by western blotting using DO1 antibody (Santa Cruz) and is shown in the lower panels.

To verify whether DNA damage-induced Id4 upregulation is dependent on endogenous mutant p53 protein, we analyzed Id4 mRNA levels in SKBr3 and SW480 cells stably transfected with a vector carrying sh-p53 interference. Id4 transcript was strongly compromised in the p53-silenced cells (Fig. 3A-B). These findings indicate that Id4 can be transcriptionally modulated by mutant p53 in response to DNA damaging agents.

**Fig. 4:**
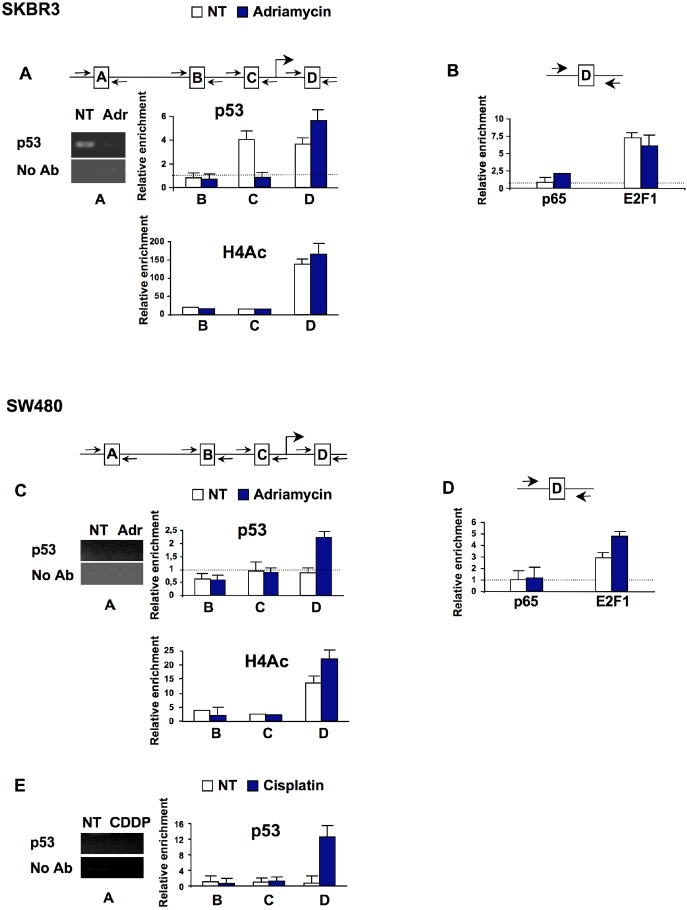
Mutant p53 associates with ID4 promoter in response to DNA damage **(A)**, **(C)** Cross-linked chromatin derived from SKBr3 and SW480 cells treated (T) or not (NT) with adriamycin (1μM) for 36h was subjected to ChIP as previously described [58], using antibodies directed against mutant p53 (sheep anti-p53 serum Ab7, Calbiochem) or acetylated histone H4 (Upstate Biotechnology, Inc.). Enrichment of the region A was analyzed by PCR (left panels) while regions B, C and D were analyzed by qPCR. The results are presented as folds over the No Ab sample (negative control). **(B)**, **(D)** Cross-linked chromatin derived from SKBr3 and SW480 cells treated with adriamycin (1μM) or cisplatin (1μg/mL) for 36h was subjected to ChIP using antibodies directed against p65 (Santa Cruz, sc-372) and E2F-1 (Santa Cruz, sc-193). The enrichment of region D was analyzed by qPCR and results are presented as folds over the No Ab. Dashed lines indicate the threshold for binding positivity.

To further evaluate the role of mutant p53 on the transcriptional control of Id4 gene expression in response to DNA damaging agents, we analyzed the *in vivo* occupancy of mutant p53 on Id4 promoter by chromatin immunoprecipitation experiments. As previously reported, we observed the recruitment of p53R175H to the A, C and D regions of Id4 promoter in untreated SKBr3 cells (Fig. 4A). In agreement with the hypermethylated status of Id4 promoter and its silenced expression, mutant p53 did not bind any of the analyzed regions of Id4 promoter in untreated SW480 cells (Fig. 4C). Upon adriamycin treatment we found that mutant p53 is recruited only to the D region of Id4 promoter in both cell lines (Fig. 4A, 4C) and this recruitment parallels a slight increase in histone H4 acetylation in that region. Since region D of Id4 promoter contains a CDE consensus where mutant p53 and E2F1 are concomitantly bound in proliferating SKBr3 cells [27], we analyzed E2F1 occupancy in that region in response to adriamycin. As shown in Fig. 4B-D E2F1 is strongly recruited to the region D of Id4 promoter in both cell lines and its binding is enhanced in SW480 cells upon adriamycin treatment, thereby suggesting a transcriptional cross-talk between mutant p53 and E2F1 in the control of Id4 expression in response to DNA damage.

**Fig. 5:**
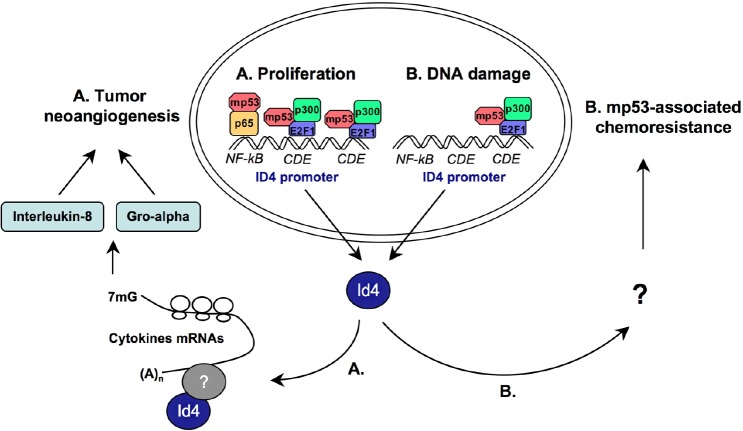
Role of mutant p53 and Id4 in breast tumor neoangiogenesis **(A)** In proliferating breast cancer cells carrying mutant p53 the protein complex mutp53/E2F1/p300 assembles on specific regions of Id4 promoter (NF-kB and CDE elements), and positively controls Id4 expression. The newly synthesized Id4 protein binds to the 3'UTR of mRNAs encoding pro-angiogenic factors, like IL8 and GRO-alpha, which contain AU-rich elements (ARE), causing their stabilization and enhancement of translation. This results in an increase of the angiogenic potential of cancer cells expressing mutant p53. **(B)** In response to DNA-damaging agents mutant p53 is specifically recruited to the downstream CDE element of Id4 promoter and transactivates its transcription in breast and colon cancer cells. The increased levels of Id4 protein probably participate to the chemoresistance of mutant p53-carrying cells.

## DISCUSSION

Id4 expression is upregulated in tumor cells carrying endogenous mutant p53 proteins upon treatment with diverse anticancer drugs. This induction is dependent on mutant p53 expression. It has recently been shown that Id1 expression is inhibited by DNA-damaging agents (camptothecin and adriamycin) in a wild-type p53-dependent manner (wt-p53) [44]. Indeed, wild-type p53 induces the transcriptional repressor DEC1, which in turn binds to Id1 promoter and represses its transcription. It appears that the impact of DNA damage on the expression of Id family members might be closely linked to the status of p53 protein. Further experimental work is needed to decipher the underlying molecular mechanisms of these events.

We found that mutant p53 is selectively recruited onto the region D of Id4 promoter in cells treated with DNA damaging agents. These findings indicate that the recruitment of mutant p53 to Id4 promoter depends on the status of the cell (proliferating or treated with DNA-damaging agents). While mutant p53 is recruited to three regions (NF-kB binding site and two CDE elements) of Id4 promoter in untreated cells [27], it binds to the downstream CDE consensus (region D) in presence of DNA-damaging agents. In cells whose Id4 expression is very low or in the presence of hypermethylation of Id4 promoter, mutant p53 does not display any binding to Id4 promoter regions in proliferating cells, while it binds to region D upon treatment with DNA-damaging agents. This suggests that reactivation of Id4 by mutant p53 in response to DNA damage agents occurs irrespective of the amount of basal Id4 and might share identical molecular events that remain to be identified.

Our data indicate that Id4 plays different roles in cancer cells carrying mutated p53 proteins. In proliferating cells Id4 plays a role in the recruitment of new blood vessels, thereby ensuring the survival and the spreading of tumor cells. Id4 might also contribute to the chemoresistance of mutant p53 tumor cells. The amounts of drugs used in the reported experiments are sub-lethal doses for SKBr3 and SW480 cells but became highly apoptotic upon depletion of mutant p53 protein [45].

Future research will be devoted to the identification of the subsets of target mRNAs that are bound by Id4 in breast cancer cells presenting wt-p53 (where Id4 is probably antitumorigenic) or mutant p53 (where Id4 is probably protumorigenic).
